# Improvements of Blood Compatibility, Drug-in-Polymer Coating Stability and Prevention of Crack Formation: Application to Drug-Eluting Stents

**DOI:** 10.3390/pharmaceutics18040506

**Published:** 2026-04-20

**Authors:** Tarek M. Bedair, Dong Keun Han

**Affiliations:** 1 Chemistry Department, Faculty of Science, Minia University, El-Minia 61519, Egypt; 2 ORANDBIO Co., Ltd., Building A Unit 410, 54, Gwangjinmal-ro, Uiwang-si 16108, Geyeonggi-do, Republic of Korea

**Keywords:** silicon nanofilament, coating stability, coating delamination, crack prevention, blood compatibility, drug-eluting stents

## Abstract

**Background/Objectives**: Commercially available drug-eluting stents still suffer from poor blood compatibility, polymer coating delamination, polymer cracking and lack of stability during and after stent implantation that led to adverse events such as stent thrombosis and in-stent restenosis. This article highlights the advantages of using silicon nanofilament (SiNf) as an interface between stent surface and drug-in-polymer coating or bloodstream. **Methods**: Thin layer of SiNf was successfully formed on the surface of Co-Cr substrate via one-step simple method. For stent applications, sirolimus-in-poly(D,L-lactide) (PDLLA/SRL) matrix was coated on control and SiNf-modified Co-Cr substrates and the stability, cracking, and long-term degradation was compared. Blood compatibility studies were also compared between control and SiNf-modified Co-Cr substrates. **Results**: The morphology of the filaments showed nanosized structures with nano-gaps between the filaments which support mechanical interlocking of PDLLA/SRL coating and enhanced the coating stability with no coating delamination whereas, the control substrate presented 97% of coating delamination. The PDLLA/SRL coating on stent platform demonstrates smooth and uniform morphology without webbing between stent struts. After stent ballooning, the control stent presented cracking and peeling of the polymer coating from the surface whereas, the SiNf-modified stent did not show any signs of these unfavorable defects. Moreover, SiNf-modified surface showed reduced fibrinogen adsorption and lower number of platelet adhesion with round shape morphology. **Conclusions**: Overall, this suggests that modifying the metallic substrates with SiNf could act as a universal coating for reinforcing the polymer coating stability, prevent coating defects that accompany stent ballooning, and improve the blood compatibility of the material surfaces that could have various applications to medical implants and devices.

## 1. Introduction

Cardiovascular diseases are considered as one of the main causes of death all over the world, especially atherosclerosis which is the deposition and accumulation of lipids, especially cholesterol around the artery walls leading to the narrowing of vascular artery [1]. Several kinds of stents, such as bare metal stent (BMS), drug-eluting stent (DES), and fully bioabsorbable stents, have been used to treat atherosclerosis [2]. Balloon-expandable stents are implanted to save the life of millions of patients every year [3]. However, BMS have shown high restenosis rate of up to 40%. It has proved its effectiveness to reduce in-stent restenosis rate found after the deployment of drug-eluting stents [4,5]. DES is made of three main components: (i) metal, (ii) polymer(s), and (iii) drug(s).

Cobalt-Chromium (Co-Cr) alloys that belong to ASTM standards have been extensively used in various biomedical implants and devices including stents [6]. Several kinds of polymers are applied to fabricate stent to control the drug release, such as non-degradable or biodegradable polymers. Biodegradable polymers demonstrated the applicability over the non-degradable because of the inflammation results from permanent contact of non-degradable polymers with tissue [7,8]. Sirolimus and paclitaxel are examples of anti-proliferative drugs used for stent fabrications as they can inhibit smooth muscle proliferation and decrease the incidence of in-stent restenosis [9].

Several strategies to introduce polymer coating on stent substrate including plasma deposition, electrospinning, layer-by-layer self-assembly, dipping, and spray coating technique have been summarized [10,11]. For polymer-based drug-eluting stents, several studies demonstrated that after stent expansion, several coating defects such as polymer cracking, detachment, and delamination form the stent surface, which led to uncontrolled the drug release, and increased the possibility of in-stent restenosis, inflammation, and/or late thrombosis [12,13,14]. According to Bedair et al., surface modification using polymer brushes (hydrophilic or hydrophobic) provided a way to improve the adhesion between stent surface and polymer coating [15,16,17]. However, these methods consume a lot of time to fabricate such brushes on the metal surface. In addition, after the degradation of biodegradable polymer, Co-Cr will be in permanent contact with the blood and soft tissue. This alloy suffers from the adsorption of blood proteins, adhesion, and activation of platelets leading to thrombosis formation [18,19]. In this regard, the need to find an easy and cheap method to fabricate a blood compatible surface and to solve the coating defects at the same time which represent challenge to fulfill our goal.

In this work, we report an easy, low cost and effective multifunctional methodology for improving blood compatibility, increasing the roughness value, increasing the surface area, enhancing the drug-in-polymer coating stability, and preventing the crack formation of polymer-based drug-eluting stents through the formation of silicon nanofilament (SiNf) on Co-Cr surfaces. This strategy could be promisingly applied for various biomedical implants and devices.

## 2. Experimental Section

**Materials.** Minitubes Co., (Grenoble, France) supplied cobalt-chromium (Co-Cr, CC, 1 × 1 cm^2^) plates. Stent-Type Co-Cr (diameter: 2.5 mm, length: 30 mm, and thickness: 110 µm) were obtained from Bioalpha Crop. (Bandar Baru Bangi, Malaysia). Methyltrichlorosilane (MTCS), sodium dodecyl sulfate (SDS), glutaraldehyde and anhydrous toluene were purchased from Sigma Aldrich (St. Louis, MO, 63103, USA). Sirolimus (SRL), and poly(D,L-lactide) (PDLLA, 75:25, 110 kDa) were purchased from LC laboratories (Woburn, MA, USA), and Lakeshore Biomaterials, Inc. (Birmingham, AL, USA), respectively. Methylene chloride, ethanol, and 1,4-dioxane were purchased from Daejung chemicals & Metals Co. (Gyeonggi-do, Republic of Korea). All the reagents were used as received without further purification.

**Pretreatment for Co-Cr substrate.** The Co-Cr samples were mechanically polished as previously described [15]. Briefly, the Co-Cr plates were mechanically grinded by silicon carbide papers followed by cloth polished using 0.3 µm alumina suspensions for 8 min each. The polished Co-Cr substrates were washed using deionized water, ethanol and acetone for 20 min each. Prior to the deposition, the Co-Cr specimens were further activated using oxygen plasma for 5 min.

**Silicon nanofilament formation on Co-Cr specimens.** The oxygen plasma treated Co-Cr samples were placed in Teflon-coated closed chamber and immersed in 200 mL of anhydrous toluene at 33–35% of humidified nitrogen (Figure S1). Then, 200 µL of MTCS was injected into the chamber using long-needle and the reaction continued for 1.5 h under 200 rpm stirring at room temperature. At the end of the reaction, the SiNf-modified substrates were taken out and washed with toluene, ethanol, and ethanol/water (1:1, *v*/*v*) three times each. The samples were dried using nitrogen gas and annealed at 120 °C for two hours. Finally, the samples were stored under vacuum until used and coded as CC-SiNf.

**Surface characterization.** The chemical functional groups on the surface of Co-Cr were studied using attenuated total reflectance-Fourier transform infrared (FT/IR-4100 instrument, ATR PRO450-S, JASCO Corporation, Tokyo, Japan). The elemental compositions of the Co-Cr and CC-SiNf surfaces were calculated using X-ray photoelectron spectroscopy (XPS; S-Probe, Surface Science Co., Plano, Texas, USA), spot size (100 µm × 100 µm), and survey scans (0–1000 eV). Static water contact angle measurements were determined using DGD Fast/60 contact angle meter (GBX, New Technologies Development, Romans-sur-Isère, France) through the addition of 2.5 µL of deionized water using micro pipette onto different areas of the substrate surface for ten times and the average values were calculated. Surface morphologies of flat and stent-type Co-Cr, CC-SiNf, and CC-SiNf-PDLLA/SRL were watched using field emission-scanning electron microscope (FE-SEM, Hitachi S4200, Tokyo, Japan). The topography and the roughness of flat Co-Cr and CC-SiNf substrates were visualized using atomic force microscope (AFM) using XE-100 (Park system, Gwacheon, Republic of Korea) under a tapping mode (10 µm × 10 µm) at room temperature. The root mean square (RMS) roughness values were calculated from the average of three spots on three different samples.

**Drug-in-polymer coating on flat and stent-type Co-Cr substrates.** A solution of PDLLA containing SRL (0.4 wt%, 60:40 wt%) was prepared by dissolving 19.9 mg of PDLLA and 13.3 mg of SRL in 8.31 g of methylene chloride/dioxane (1:1, *v*/*v*) until a homogenous solution was obtained. The prepared solution was the ultrasonic spray coated on flat and stent-type Co-Cr substrates as previously mentioned [20,21]. Finally, the PDLLA/SRL coated on Co-Cr substrates were dried in vacuum chamber overnight and coded as CC//PDLLA/SRL, and CC-SiNf//PDLLA/SRL, respectively.

**In vitro coating stability under circulating buffer solution.** Both CC//PDLLA/SRL and CC-SiNf//PDLLA/SRL flat substrates were inserted into 10 mm diameter silicon tube. Rate controller apparatus (100 rpm) circulated a buffer solution of PBS (pH 7.4) for 30 days. The samples were taken out of the silicon tube, washed with deionized water three times, and dried under vacuum. The surface morphology of PDLLA/SRL coating after circulation test was examined using FE-SEM.

**In vitro stent balloon inflation.** Both CC//PDLLA/SRL and CC-SiNf//PDLLA/SRL stents were mounted onto angioplasty balloon and dilated to 3 mm at 10 atm pressure for 30 s. The morphologies of the expanded PDLLA/SRL coated stents were studied using FE-SEM.

**Fibrinogen adsorption.** The control and SiNf-modified Co-Cr substrates were prewashed with phosphate-buffered saline (PBS) solution followed by immersion in fibrinogen solution with a concentration of 100 µg/mL for 1 h at 37 °C. The samples were washed with 1 mL of PBS solution three times. For quantitative analysis, 400 μL of 5% SDS solution was used to detach the adsorbed fibrinogen overnight. Then, 100 μL of the SDS/fibrinogen solution was mixed with 100 μL of Micro-BCA solution in a 96-well plate. Finally, the plate was kept at 37 °C for 2 h followed by UV measurements at 562 nm. For qualitative analysis, fluorescent fibrinogen was used following the same protocol and the images were captured using fluorescence microscopy (Olympus, Tokyo, Japan).

**Platelet adhesion and morphology.** To study the effect of superhydrophobic property of SiNf on the platelet adhesion, the control Co-Cr and CC-SiNf substrates were sterilized under UV for 1 h. The number of platelets in platelet-rich plasma was adjusted to be 1 × 10^7^ cells/mL by the addition of platelet-poor plasma. The samples were dipped in 1 mL of platelet suspension at 37 °C for 1 h and the samples were carefully washed with PBS solution three times. For lactate dehydrogenase (LDH) assay, the samples were lysed using 1 mL of 2% Triton X-100 at 37 °C for 15 min. Then, 100 μL of each sample was mixed with equal volume of LDH kit (Takara Bio, Inc., San Jose, CA, USA) reagent in the 96-well plate. Then, the 96-well plate was kept at 37 °C for 1 h and UV absorbance was measured at 490 nm. For SEM analysis, the adhered platelets on both substrates were fixed with 1 mL of 2.5% glutaraldehyde solution for 1 h. Finally, they were dehydrated sequentially with 50, 60, 70, 80, 90, 95, and 100% ethanol solution. Thereafter, the samples were vacuum dried and then spattered with platinum for 60 s to perform the SEM analysis using FE-SEM.

**Statistical analysis.** Data are expressed as mean ± standard deviation and considered statistically significant (*) when *p* < 0.05, (**) when *p* < 0.01, and (***) when *p* < 0.001 as determined by one-way ANOVA followed by Tukey’s post hoc test using IBM SPSS statistics 20 software.

## 3. Results and Discussion

### 3.1. Fabrication of Silicon Nanofilament on the Co-Cr Surface

**Figure 1:**
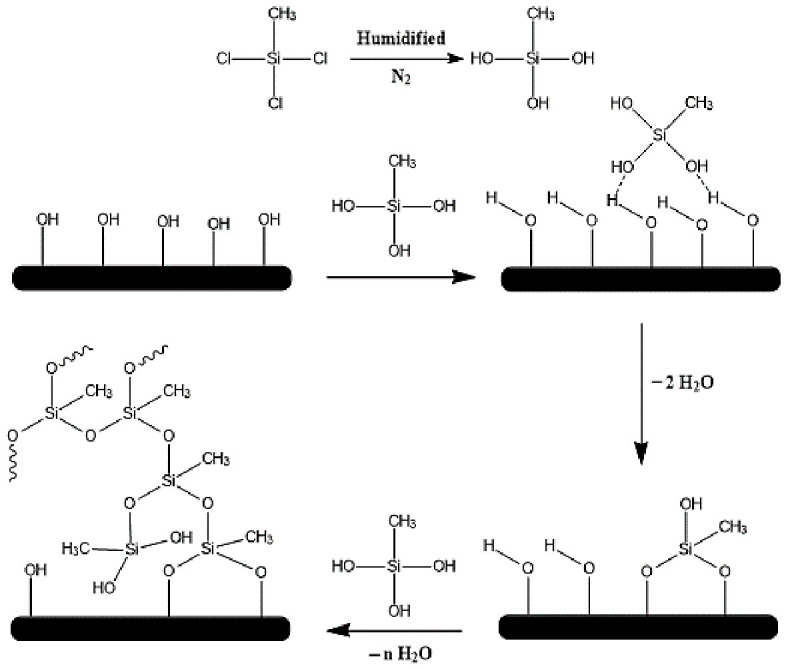
Schematic representation for the reaction of MTCS and surface-activated Co-Cr substrate.

Oxygen plasma has been used to activate metal surfaces and produce several groups such as hydroxyl (-OH), peroxyl (C-O-O-), carbonyl (C=O), carboxyl (O=C-OH), or carbonate (O-C(O)-O) groups on their surfaces [22,23]. MTCS is non-fluorinated organosilicon monomers which can undergo silylation with hydroxyl groups on material surfaces to form hydrophobic structures [24]. Once MTCS is injected into the humidified toluene solution, the chlorosilane (Si-(Cl)_3_) groups are hydrolyzed easily to give silanol groups (Si-(OH)_3_) [25]. The silanol could be initially assembled on the activated Co-Cr surface through hydrogen bonds. As time proceeds, condensation reactions between hydroxyl and silanol groups occurred which leads to the formation of covalent bonds and the growth of silicon nanostructure. It is worth to note that the relative humidity inside the chamber can govern the formation of particles or filament like morphology [26]. According to previous reports, it was found that 35 ± 5% of relative humidity is the optimal condition for the formation of nanofilament morphology [27]. The proposed mechanism of interaction between the activated Co-Cr and MTCS is clearly presented in Figure 1.

### 3.2. Surface Characterization of Silicon Nanofilament

**Figure 2:**
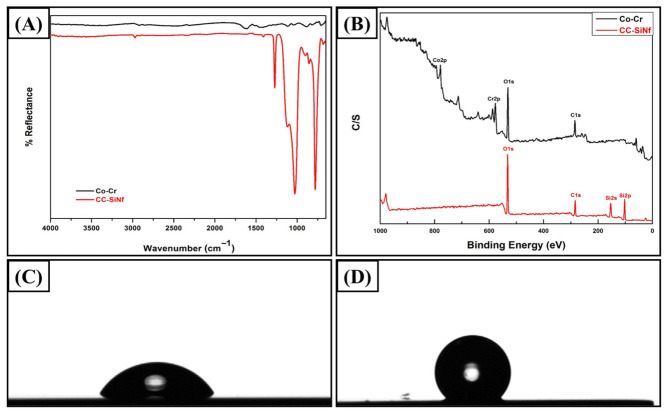
(**A**) ATR-FTIR scan (650–4000 cm^−1^) (control Co-Cr (black color) and CC-SiNf substrates (red color)), (**B**) wide scan XPS spectra (0–1000 eV) (control Co-Cr (black color) and CC-SiNf substrates (red color)), (**C**) water droplet image on control Co-Cr substrate, and (**D**) water droplet image on CC-SiNf substrate.

To study the chemical structure of thin film on the metal substrates, ATR-FTIR is a commonly used analysis technique to determine the functional groups on the surface for a depth of up to 5 μm. Figure 2A shows the ATR-FTIR spectrum of control Co-Cr (black color) and SiNf-modified Co-Cr substrates (red color). The control Co-Cr plate did not show any absorption peaks in FTIR spectra as reported [17]. On the other hand, the CC-SiNf samples showed several characteristic peaks of polymethylsilsesquioxane structure. Two peaks appeared at 1272 and 779 cm^−1^, which can be ascribed to asymmetric stretching vibration of (OSi-CH_3_) deformation vibrations and bending vibration of (O-Si-O) bond of the siloxane compound, respectively [25]. The peaks at 1117 and 1026 cm^−1^ were attributed to Si-O-Si vibration of siloxane compound. In addition, two absorption peaks appeared at 2950 and 2850 cm^−1^, which can be attributed to asymmetric and symmetric C-H stretching vibrations, respectively [28,29]. To verify the chemical compositions of SiNf on Co-Cr substrate, X-ray photoelectron spectroscopy (XPS) was performed. Figure 2B shows the wide scan XPS survey of control (black color) and SiNf-modified Co-Cr substrates (red color). Table 1 summarizes the elemental compositions of the surface before and after modification by silicon nanofilament. The bare Co-Cr sample showed the constituent peaks of the alloy (i.e., Co, Cr, and O, in addition to carbon results from contaminations) [30,31]. On the other side, CC-SiNf samples presented new peaks that appeared at 153.2 and 102 eV, which were attributed to Si2s and Si2p, respectively. In addition, the contents of the (O) elements increased, the contents of the (C) elements dramatically decreased, and both of (Co) and (Cr) elements disappeared. Moreover, the C/Si ratio for the CC-SiNf was 1.05, which is close to the theoretical value of MTCS (C/Si = 1). These results further indicate that MTCS were successfully grafted on Co-Cr surface.

The wettability of various substrates can be easily determined through the measurements of water contact angle with the surface. Figure 2C,D shows the water contact angle of the Co-Cr substrate before and after modification using silicon nanofilaments. The water contact angle of the flat Co-Cr substrate was 58 ± 1° (Figure 2C, Table 1), whereas after oxygen-ion-beam treatment, the water contact angle dropped to 11^o^ due to the formation of copious amounts of hydroxyl groups on the metal surface that increase the surface energy. After the formation of SiNf, the water contact angle increased to 137 ± 3^o^ due to the hydrophobic nature of MTCS as well as the surface topography (Figure 2D, Table 1) [32]. Similarly, the reaction of MTCS on the surface of sodium alginate/poly(vinyl alcohol)/palygorskite film improved the hydrophobicity of the surface to 112° [29]. A very close value of water contact angle at 131° was measured when nanofibers of MTCS were formed on carboxymethylated cellulose films [25].

**Figure 3:**
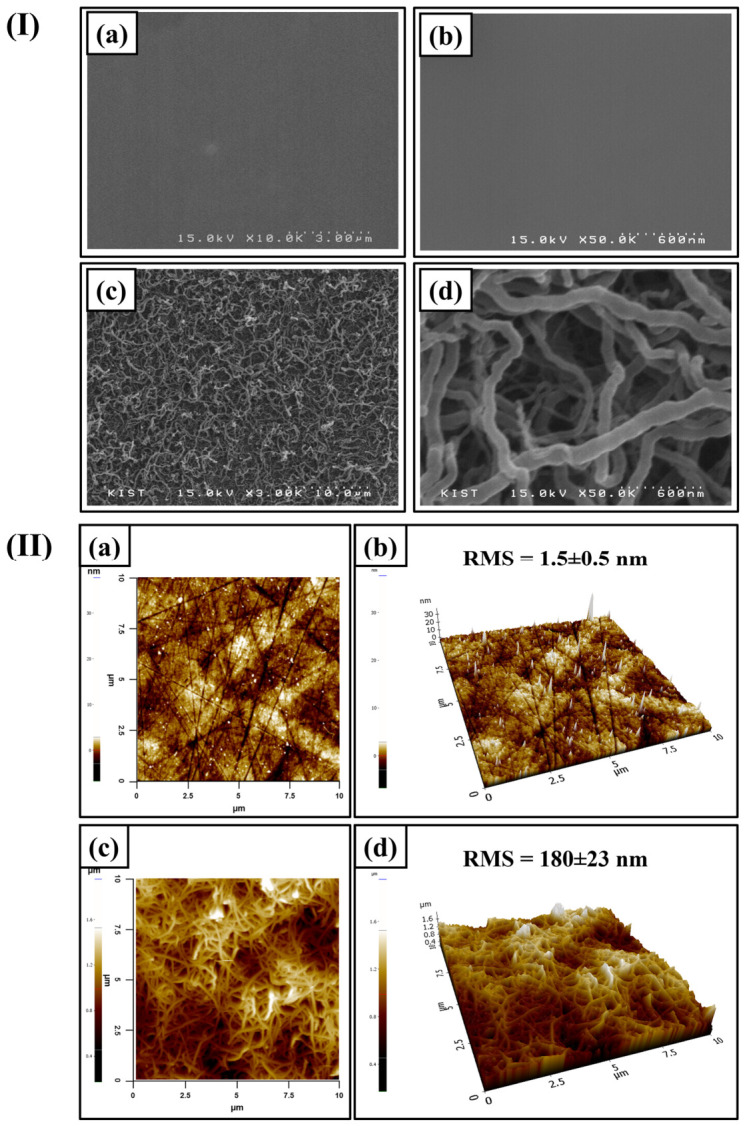
(**I**) SEM morphologies for (**a**) low magnification of control Co-Cr substrate, (**b**) high magnification of control Co-Cr substrate, (**c**) low magnification of CC-SiNf substrate, and (**d**) high magnification of CC-SiNf substrate. (**II**) 2D and 3D AFM topography as well as the RMS roughness values for: (**a**,**b**) control Co-Cr substrate; (**c**,**d**) CC-SiNf substrate.

Scanning electron microscopy (SEM) is a common technique to determine the change in surface morphology of various substrates after modifications. The differences in the surface morphology and topography of the Co-Cr substrate due to SiNf formation are displayed in Figure 3I,II, respectively. The flat Co-Cr substrate displayed smooth surface (Figure 3I(a,b)). The CC-SiNf surface showed nanofiber morphology with diameter ranging between 60 and 100 nm (Figure 3I(c,d)). Interestingly, nanopores or nanogaps were observed between the interconnected filaments. Atomic force microcopy (AFM) is a useful technique for qualitatively clarifying the surface topography and quantitatively determining the surface roughness value. Figure 3II(a,b) represents the 2D and 3D images for the flat Co-Cr substrate, respectively, with 1.5 ± 0.5 nm roughness value. On the other hand, the 2D and 3D images of the CC-SiNf substrates were shown in Figure 3II(c,d), respectively, and the roughness value was 180 ± 23 nm. This demonstrated that the formation of SiNf on any flat substrate increases the roughness value and consequently greatly enhances the surface area of the substrate [27], which could support the upper polymeric layer for stent application. The thickness of the nanofilaments formed on Co-Cr substrate was determined using cross-section of silicon wafer at the same experimental conditions. It was found that the thickness was in nano scale which was 436 ± 46 nm (Figure S2).

Based on analyzing the results of ATR-FTIR, XPS, water contact angle, SEM and AFM, the Co-Cr alloy was successfully modified by a hydrophobic nanofilament thin layer with greater roughness.

### 3.3. Characterization of Drug-in-Polymer Coating

**Figure 4:**
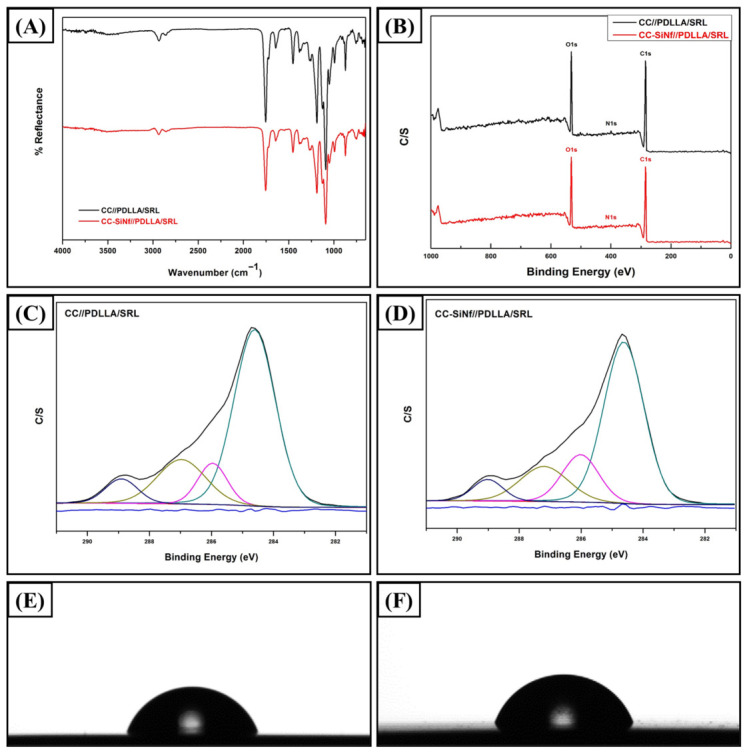
(**A**) ATR-FTIR spectra of the drug-in-polymer (i.e., PDLLA/SRL) coating (650–4000 cm^−1^) (control Co-Cr (black color) and CC-SiNf substrates (red color)), (**B**) wide scan XPS spectra (0–1000 eV) (control Co-Cr (black color) and CC-SiNf substrates (red color)), (**C**) C1s narrow scan of CC//PDLLA/SRL, (**D**) C1s narrow scan of CC-SiNf//PDLLA/SRL, (**E**) water droplet image on CC//PDLLA/SRL substrate, and (**F**) water droplet image on CC-SiNf//PDLLA/SRL substrate.

Ultrasonic spray coating instrument is a famous apparatus to coat the drug-in-polymer matrix on various shape platforms including stents [33,34]. Figure 4A represents the ATR-FTIR spectra of the control Co-Cr (black color) and CC-SiNf substrates (red color) after being coated with a matrix of PDLLA and SRL. The characteristic peaks of both PDLLA and SRL were clearly observed at 1755, 1722, and 1644 cm^−1^, which are attributed to stretching vibrations of C=O (PDLLA), stretching vibrations of C=O (SRL), and stretching vibrations of C=C (SRL), respectively. In addition, two bands at 1188, and 1091 cm^−1^ are attributed to backbone of PDLLA ester groups [35,36]. Moreover, several peaks were found at 2935, 2854, 1453 and 1380 cm^−1^, which are assigned to asymmetric C-H stretching, symmetric C-H stretching, -CH_2_ bending, and -CH_3_ bending vibrations, respectively. The presence of these peaks suggested that a mixture of PDLLA and SRL was successfully coated on the surface of metal stent. Figure 4B represents the wide scan XPS analysis for PDLLA/SRL coated on control Co-Cr (black color) and CC-SiNf substrates (red color). Three main peaks were observed at ~284.6, 400, 532 eV, which are attributed to C1s, N1s, and O1s, respectively. Interestingly, the Co2p, Cr2p, and Si2p disappeared from the spectra, which proved that the coating was uniform along the surface without debris. Table 1 summarizes the atomic percentage of the PDLLA/SRL matrix surface. For the PDLLA/SRL coated on CC-SiNf samples, the N1s shows lower value compared to the PDLLA/SRL coated on flat Co-Cr substrate, which most probably related to the incorporation of SRL between the silicon nanofilaments. In addition, the water contact angle of the SRL-in-PDLLA coating represents more hydrophobic property with water contact angle of 72.4° vs. 70.4° of the flat surface as shown in Figure 4E and Figure 4F, respectively.

Figure S2a–d shows the SEM images of the SiNf, small amount of drug-in-polymer matrix ultrasonic spraying on SiNf-modified silicon wafer substrate, and the cross-section of the coating on SiNf substrate. From these images, it is proved to be an interlocking mechanism that takes place during the coating as the polymer penetrates inside the interconnected nanofilaments and fills the nanopores as clearly shown in Figure S2c,d. According to our experiments, the thickness of the drug-in-polymer coating on flat CC-SiNf substrate was determined from the cross section to be 1.94 ± 0.13 µm (Figure S3).

### 3.4. In Vitro Stability Under Circulating Buffer

**Figure 5:**
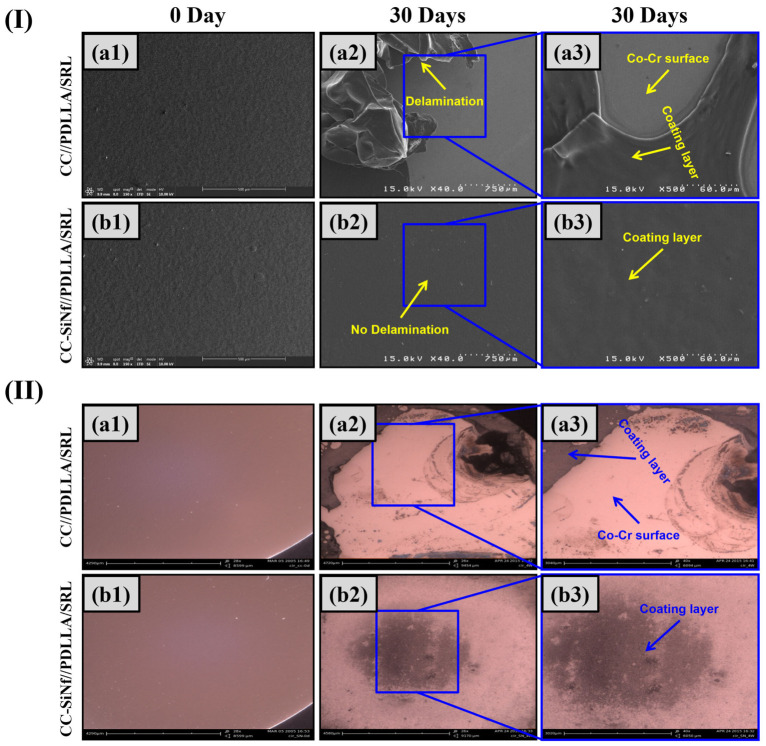
(**I**,**II**) SEM and optical microscopy images for PDLLA/SRL coating on control Co-Cr (**a1**–**a3**) and CC-SiNf substrates (**b1**–**b3**), respectively, at zero day (**a1**,**b1**) and after 30 days of circulation (**a2**,**a3**,**b2**,**b3**).

The stability of SRL-in-PDLLA coating on control Co-Cr flat and CC-SiNf substrates were studied under circulating physiological buffer that mimic heart-pumping system in our body. Figure 5I,II represent the SEM and light microscopy images of the SRL-in-PDLLA coating at zero day and after 30 days in circulating buffer. At zero day, the morphology of the drug-in-polymer matrix on flat Co-Cr and CC-SiNf substrates, presented smooth and uniform coating Figure 5I(a1,b1), respectively. Interestingly, the CC-SiNf substrate did not show any filament morphology after coating, which means that the nano-holes were fully and uniformly filled with the PDLLA/SRL coating with a smooth upper surface. At 30 days, for control Co-Cr substrate, the coating morphology shows unfavorable phenomena of coating delamination for up to 97% after the circulation of buffer solution (Figure 5I(a2,a3), II(a2,a3)). On the other side, the CC-SiNf samples did not show any sign of delamination during circulating buffer which proved that the coating was stable even after pumping pressure (Figure 5I(b2,b3), II(b2,b3)). Several reports correlate the surface modification using polymer nano-brushes and the enhancement of interfacial adhesion and improvement of coating stability [15,20]. Another report prove that the interfacial modification between metal and polymer could be improved due to modification using silicon materials owing to the diffusion of silicon in to the polymer coating [37]. This could be the same reason for the enhancement of coating stability of the polymer layer on the CC-SiNf substrates. Another proposed mechanism known as mechanical interlocking in which the drug-in-polymer matrix (the key) penetrates into the nanopores of SiNf on Co-Cr substrate (the lock), as clearly shown in Figure S2d, which increased the adhesion greatly [38,39,40]. When coating porous magnesium alloy with polylactide multilayer, a mechanical locking is observed [41]. As reported, porous polymer coatings show excellent adhesion on planar and curved substrates due to interfacial mechanical interlocking mechanisms [42]. Moreover, it is suggested that the polymer could physically react tightly with the methyl groups present in the chemical structure of MTCS through hydrophobic interactions. Similar study demonstrates a hydrophobic interaction between hydrophobic SRL drug and hydrophobic 3D porous nano-networked silica film coating for stent application [43]. Altogether, the enhancement of the polymer coating stability could be due to a series of reasons such as the diffusion of silicon in polymer coating, hydrophobic interaction with the methyl group present on the surface, and the interlocking mechanism.

### 3.5. In Vitro Balloon Inflation

**Figure 6:**
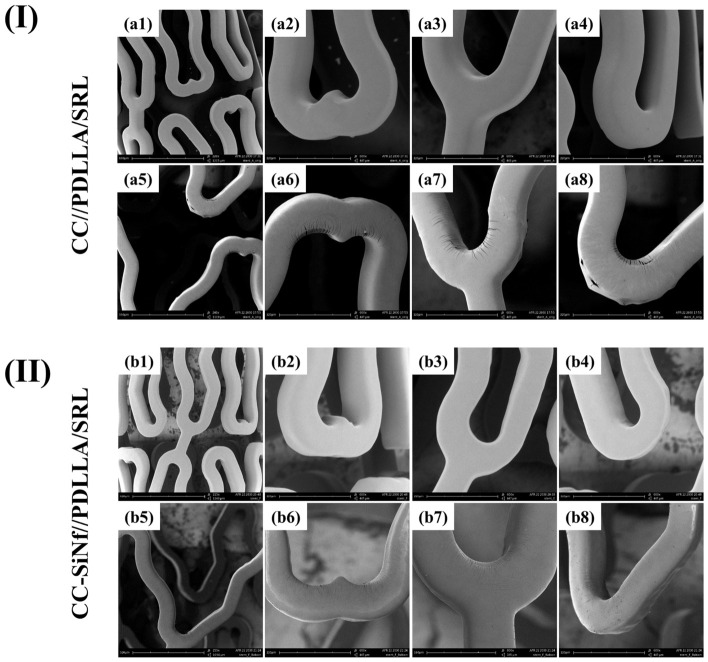
SEM morphologies of PDLLA/SRL coating on: (**I**) control Co-Cr stent—before ballooning (**a1**–**a4**), and after ballooning (**a5**–**a8**), respectively; (**II**) SiNf-modified Co-Cr stent—before ballooning (**b1**–**b4**), and after ballooning (**b5**–**b8**).

For the applicability of our strategy in the stent manufacturing, SiNf stents were fabricated and fully coated using a drug-in-polymer matrix and finally expanded using balloon following the standard protocol. Figure 6 represents SEM images of the SRL-in-PDLLA matrix coated on control and SiNf-modified stents. Before stent ballooning, the coating on both samples showed smooth and uniform morphology without webbing between stent struts (Figure 6I(a1–a4),II(b1–b4)). Coating defects after ballooning represent serious drawbacks for the failure of stent after in vivo implantation, which increase the risk of stent thrombosis and in-stent restenosis that could lead to death [12,17]. After stent ballooning, the morphology of the coating showed different behavior. For control stent, various coating defects were observed after stent expansion such as cracking, delamination, and detachments from the stent surface (Figure 6I(a5–a8)). On the other hand, the SiNf-modified stent prevents such kinds of coating defects after expansion (Figure 6II(b5–b8)). Similarly, PDLLA coating on magnesium alloy showed significant cracking and significantly reduced charge transfer resistance in the regions of strain concentration [44]. The presence of polymer nano-coupled interlayer on the surface of Co-Cr stent enhanced the ability to prevent crack formation of poly(lactide-co-glycolide) [17]. It was also found that ion-beam treatment for polylactide thin layer results in the formation of nanopores which support the upper drug-in-polymer matrix coating and prevents cracking during stent ballooning [33]. Similarly, nanoporous coating demonstrates excellent adhesion with polymer substrate and the ability to withstand repeated bending on two- or three-dimensional surfaces [45]. Furthermore, an interfacial anchoring mechanisms were established between porous polymer coatings and planar and curved substrates [42]. In addition, the presence of nanoporous coating layer on stent surface can prevent the crack formation of the top polymer layer against expansion force during balloon inflation [33]. This demonstrated that SiNf at the stent surface could promisingly prevent the coating defects and consequently open a window to finally manufacture safe biodegradable polymer-based drug-eluting stents without any side effects. The main reason for the prevention of polymer cracking could possibly be due to the formation of interlocking between the polymer coating and the nanoholes between nanofilaments (Figure S2b–d). In addition, the hydrophobic interactions between the methyl group found in the chemical structure of polymethylsilsesquioxane nanolayer and the drug-in-polymer coating could be another reason.

### 3.6. Blood Compatibility Study

**Figure 7:**
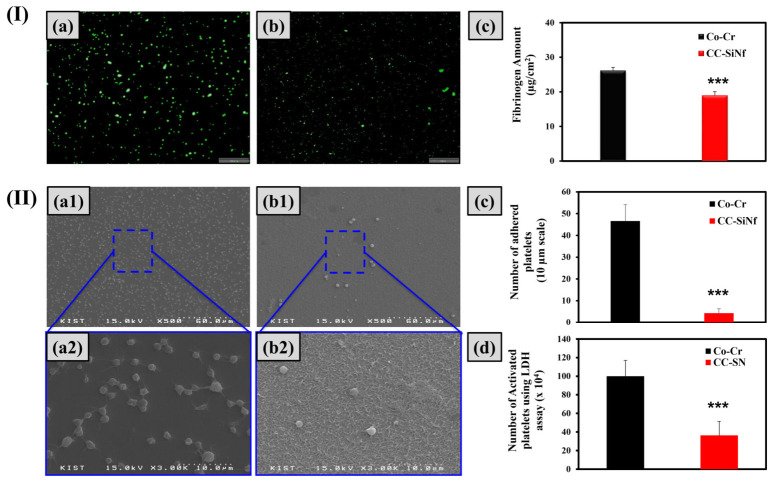
(**I**) Fibrinogen adsorption test: (**a**,**b**) fluorescence image of adsorbed fibrinogen on flat Co-Cr, and CC-SiNf substrates (scale bare 200 µm), and (**c**) the amount of adsorbed fibrinogen on the surface of substrates using micro-BCA assay. (**II**) Scanning electron microscopy images for the adhered platelets: (**a1**,**a2**) flat Co-Cr substrate, and (**b1**,**b2**) CC-SiNf substrate (scale bare 60, and 10 µm, respectively), (**c**) the number of adhered platelets from SEM images (scale bare 10 µm), and (**d**) the number of activated platelets using LDH assay (*** *p* < 0.001).

The first stage of interaction between material surface and blood is the adsorption of nonspecific protein, i.e., fibrinogen, that leads to undesirable results [46,47]. The adsorption of tinny amounts of fibrinogen can initiate a series of events including platelet adhesion, and activation which lead to coagulation and thrombus formation [48]. The amount and the conformation change in the adsorbed protein on the surface are very important to evaluate blood compatibility. Figure 7I(c) shows the fibrinogen amounts on the Co-Cr surface before and after SiNf modifications. The flat Co-Cr substrate presented higher affinity toward fibrinogen adsorption as compared to CC-SiNf substrate (*** *p* < 0.001). This demonstrated an improvement of blood compatibility after the formation of SiNf on the surface of Co-Cr substrate.

To ensure the long-term hemocompatibility of SiNf substrate, an in vitro platelet adhesion experiment for the control and modified samples was tested. This study plays an important role in determining the blood compatibility of any blood-contacting materials [49]. Thrombosis formation, which causes potential danger and lead to death, is a sequence of physiological reactions that started with platelet adhesion, then aggregation and spreading [50]. The shape of platelets, i.e., round, dendritic, spreading dendritic, and fully spreading forms, determine the activation stages of the platelets [51]. Figure 7II(a,b) shows the SEM images of the adhered platelets on the unmodified and modified Co-Cr surface after in vitro platelet adhesion test. The number and activity of platelets adhered to the CC-SiNf substrate was lower and highly significant than that on flat Co-Cr surface (*** *p* < 0.001) (Figure 7II(c,d)). In addition, there was difference in adhered platelet morphology with round shape on CC-SiNf substrate (Figure 7II(b1,b2)) and pseudopodium on control Co-Cr substrate (Figure 7II(a1,a2)) which indicates that the CC-SiNf substrates have better blood compatibility. Several reports have presented a relationship between surface wettability and platelet adhesion [52,53]. The laser-treated polydimethylsiloxane (PDMS) rubber which produce superhydrophobic property reduced the platelet number and activation as compared to untreated PDMS [53]. According to Zhou et al., the micro-patterned textured surface demonstrated lower platelet adhesion and activation as compared to flat surface, especially when the surface was superhydrophobic [37]. Our study proves that the formation of a thin layer of SiNf reduced the number and activity of adhered platelets possibly for two reasons. Firstly, the hydrophobic nature of SiNf substrate, and secondly, the pressure of the trapped gas inside the nano holes could possibly prevent the platelets from adhering to the surface.

To highlight the novelty of the proposed SiNf-modified surface, a comparison with commonly reported surface modification strategies for drug-eluting stents is presented in Table 2. Traditional polymer coatings suffer from mechanical instability such as cracking and delamination, which may lead to uneven drug release and adverse vascular responses such as in-stent restenosis and thrombosis. Compared to conventional approaches and previously reported methods for surface modifications, the SiNf-layer on Co-Cr stent provides improved polymer coating stability and reduced crack formation, which are critical for achieving controlled drug delivery and long-term in vivo performance. Furthermore, the hemocompatibility of SiNf-modified stent was improved compared to control stent through significantly reduced fibrinogen adsorption and platelet adhesion and activation. This technique is simple, cost-effective, reproduceable and safe.

Based on the results obtained in this study through the improvement of coating stability, prevention of crack formation, and favorable hemocompatibility, the SiNf-modified stents are expected to exhibit enhanced long-term in vivo performance, particularly in terms of uniform drug release, reduced thrombogenicity, and potentially lower restenosis risk. The previous report demonstrated a relationship between improved coating stability and reduced in-stent restenosis area, likely due to improved surface properties and drug release behavior [20].

At the end of this study, further studies are required, such as in vitro smooth muscle toxicity, to provide additional validation of the safety profile of the developed stents, hemolytic activity assay to provide a more comprehensive evaluation of blood compatibility, and in vivo in-stent restenosis evaluation using suitable animal model which will be addressed in our future work.

## 4. Conclusions

In this study, we have presented multifunctional property of fabricating a thin layer of SiNf on the surface of stent substrate. The SiNf could act as a pocket for drug-in-polymer matrix and consequently improve the drug-in-polymer coating adhesion ability with metal surfaces, which in turn enhances the stability of such layer on metal stent surface even under circulating buffer. Moreover, it could also prevent coating defects such as cracking, delamination, and detachment of the coating from metallic stents after balloon expansion. The hydrophobic property of the SiNf could effectively reduce fibrinogen adsorption, and platelet adhesion and activation to a higher extent leading to the improvement of blood compatibility. It is expected that the modification of metallic substrates using SiNf could be promisingly applied to polymer-coated metal stents and blood-contacting materials for biomedical implants and devices.

## Figures and Tables

**Table 1:** XPS atomic concentrations as well as the water contact angle for control Co-Cr, CC-SiNf, CC//PDLLA/SRL, and CC-SiNf//PDLLA/SRL substrates.

Samples	XPS Atomic Concentration (%)						WCA (Degree)
	C1s	N1s	O1s	Si2p	Cr2p	Co2p	
**Co-Cr substrate**	32.22	0	47.04	2.72	13.10	4.92	58 ± 1°
**CC-SiNf substrate**	28.01	0	45.41	26.58	0	0	137 ± 3°
**CC//PDLLA/SRL**	77.67	1.13	21.20	0	0	0	70.4 ± 0.2°
**CC-SiNf//PDLLA/SRL**	76.65	1.03	22.32	0	0	0	72.4 ± 1.2°

**Table 2:** Comparison of surface modification strategies for polymer-coated drug-eluting stents.

Strategy	Mechanism	Advantages	Limitations	Ref.
Conventional polymer coating for DESs (SRL/PDLLA)	Drug embedded in polymer layer.	Controlled drug release. Clinically proven.	Delamination and crack formation. In-stent restenosis. Late thrombosis. Further optimization of parameter settings.	[44,54]
Polymer nanobrushes-modified surfaces	Nanocoupling through entanglement and hydrophobic interaction.	Improved polymer adhesion. Sustained drug release. Prevented coating delamination. Reduced in-stent restenosis.	Modification takes long time. Several steps. No blood compatibility studies.	[15,18,20]
Dual layer coating on porous substrate	Interfacial physical Interactions and entanglements between polymers.	Improved degradation morphology. Controlled drug release. Prevented polymer cracking.	No blood compatibility. No in vivo studies.	[33]
Electropolishing pretreatment and direct plasma amination	Formation of thin chromium-rich oxide layer.	Highest corrosion resistance. Best mechanical stability. Highest amination efficiency. Good hemocompatibility and biocompatibility.	No study for polymer coating DESs. No in vivo animal study.	[55]
Microporous/micro-nanostructured surfaces	Surface roughness and porosity act as drug reservoirs.	Increased surface area. Improved drug loading. Improved hemocompatibility. Controlled drug release.	Limited mechanical durability. Potential non-uniform coating. No in vivo animal study.	[56]
Silicon-based nanostructures	High surface area nanostructure.	Controlled drug loading. Good biocompatibility.	No study for polymer coating DESs. Limited clinical translation.	[43]

## Data Availability

The original contributions presented in this study are included in the article/Supplementary Material. Further inquiries can be directed to the corresponding authors.

## Supplementary Materials

The following supporting information can be downloaded at https://www.mdpi.com/article/10.3390/pharmaceutics18040506/s1. Figure S1. Schematic representation for the formation of silicon nanofilament (SiNf) on Co-Cr substrates. Figure S2. SEM images of SiNf grown on silicon wafer substrate: (a) control SiNf, (b) PDLLA/SRL coating (0.03 ml solution), (c) PDLLA/SRL coating (0.05 ml solution), and (d) cross-section of PDLLA/SRL coating morphology (0.05 ml solution). Figure S3. The thickness of SiNf on silicon wafer: (A) cross-section image of the SiNf nanolayer (Scale bare 2 µm), and (B) cross-section image after being coated with PDLLA/SRL (Scale bare 5 µm).
