# Supplementary material for: Improvements of Blood Compatibility, Drug-in-Polymer Coating Stability and Prevention of Crack Formation: Application to Drug-Eluting Stents
Source: Pharmaceutics. 2026 Apr 20;18(4):506. doi: 10.3390/pharmaceutics18040506 (PMC13120645; doi:10.3390/pharmaceutics18040506)

Supplementary Materials

Improvements of Blood Compatibility, Drug-in-Polymer Coating Stability and Prevention of Crack Formation: Application to Drug-Eluting Stents

Tarek M. Bedair[1,*], and Dong Keun Han[2,*]

[1] Chemistry Department, Faculty of Science, Minia University, El-Minia 61519, Egypt

[2] ORANDBIO Co, Ltd. Building A Unit 410, 54, Gwangjinmal-ro, Uiwang-si, Geyeonggi-do 16108, Republic of Korea

Figure S1. Schematic representation for the formation of silicon nanofilament (SiNf) on Co-Cr substrates.

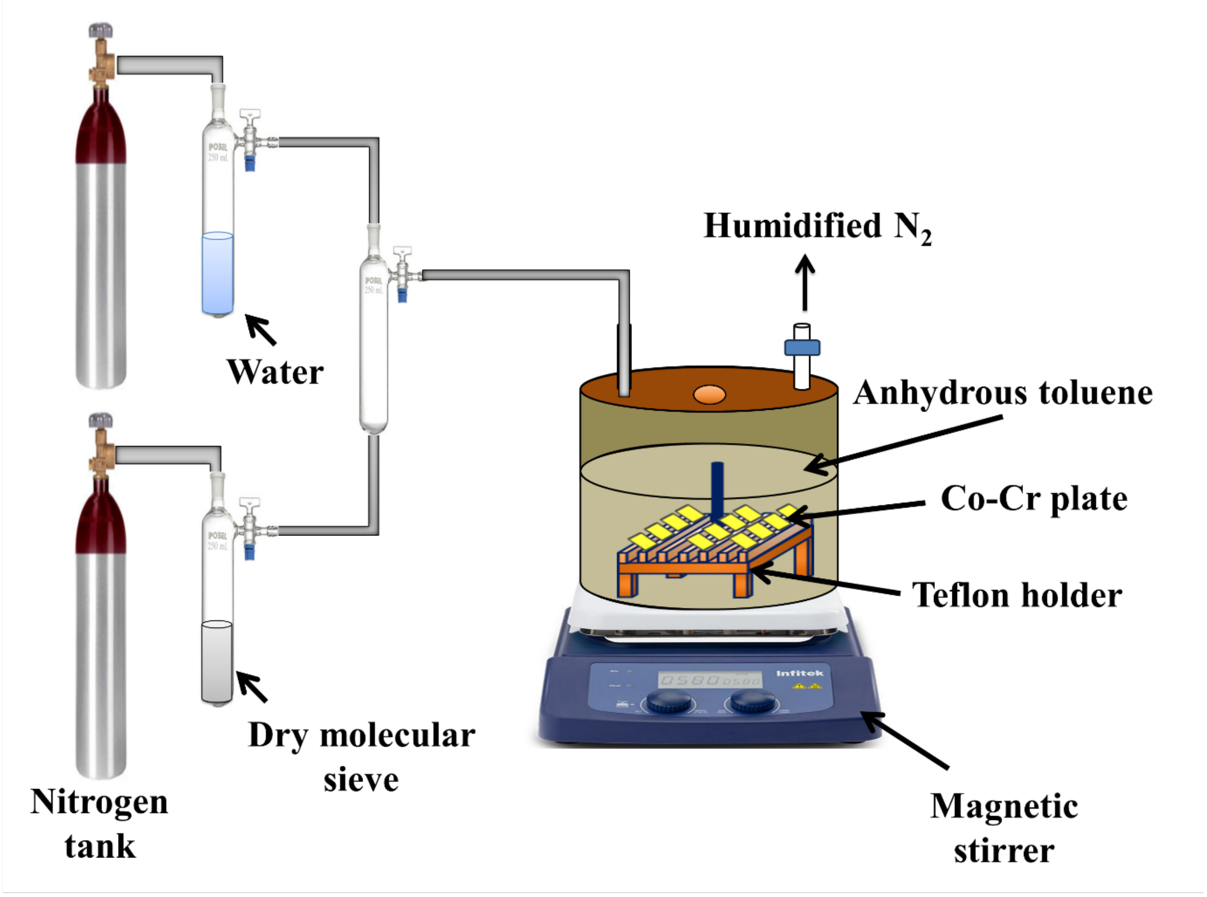

Figure S2. SEM images of SiNf grown on silicon wafer substrate: (a) control SiNf, (b) PDLLA/SRL coating (0.03 ml solution), (c) PDLLA/SRL coating (0.05 ml solution), and (d) cross-section of PDLLA/SRL coating morphology (0.05 ml solution).

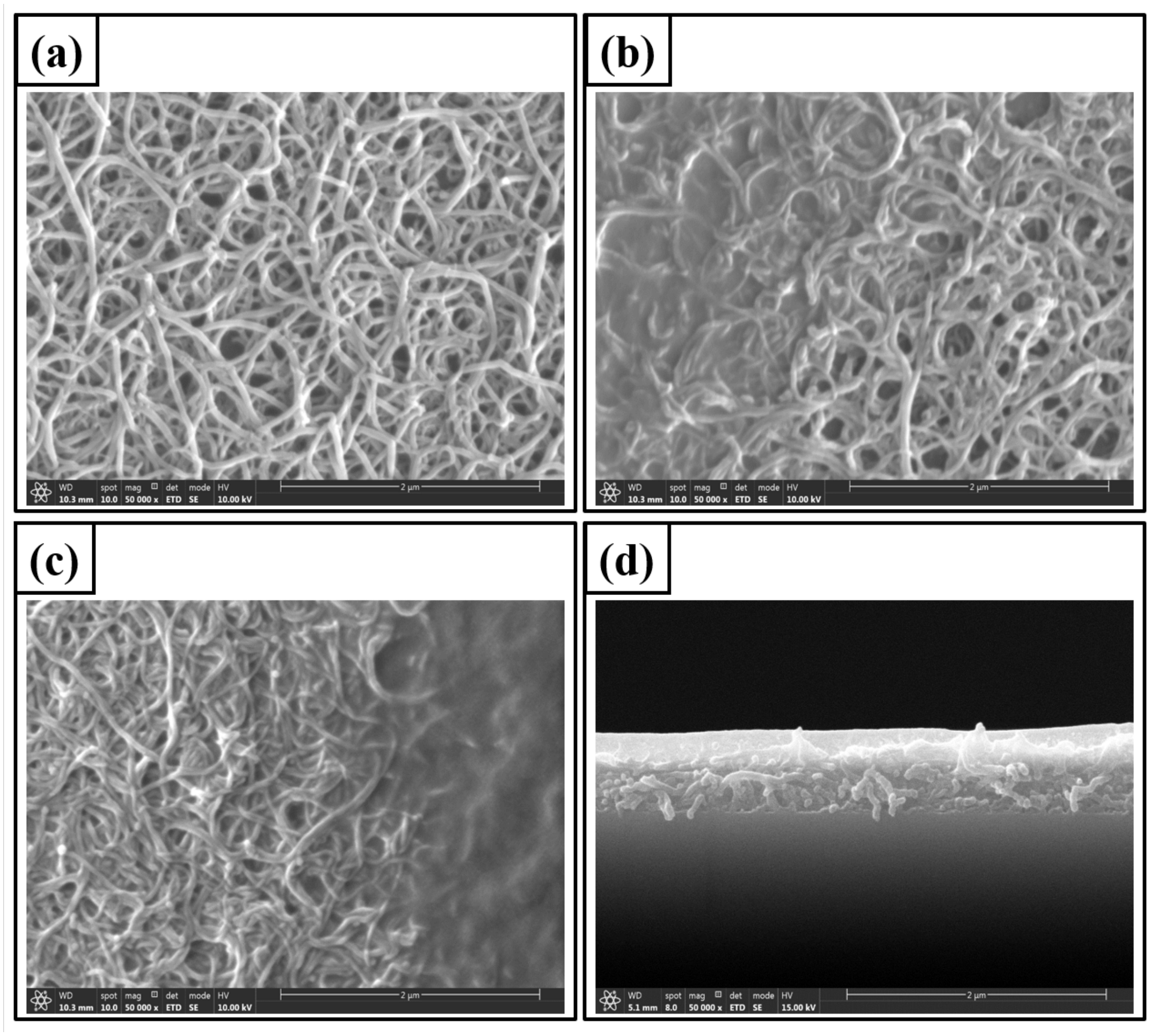

Figure S3. The thickness of SiNf on silicon wafer: (A) cross-section image of the SiNf nanolayer (Scale bare 2 µm), and (B) cross-section image after being coated with PDLLA/SRL (Scale bare 5 µm).

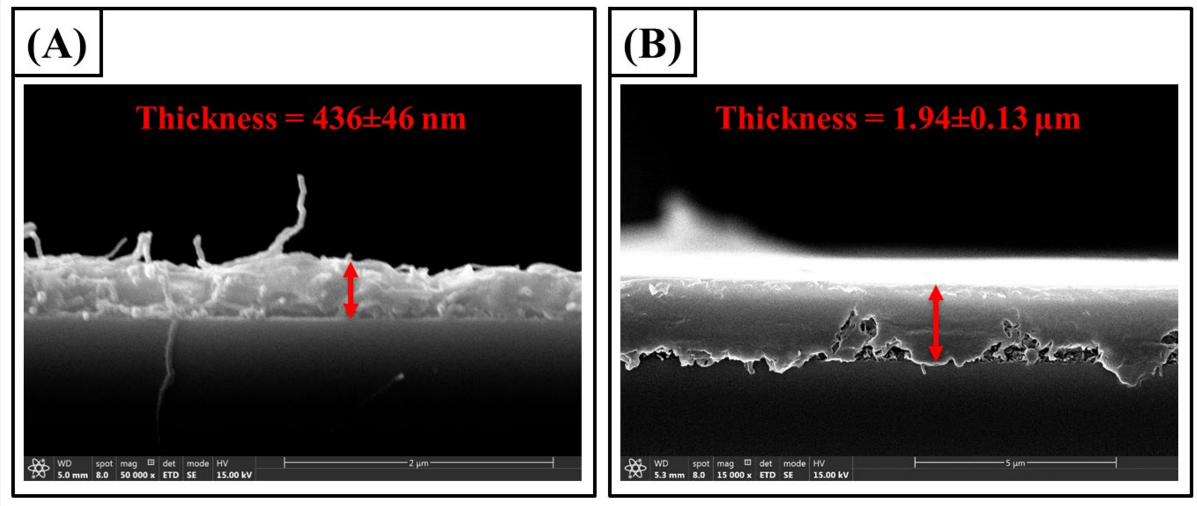

Supplement: Supplementary file 1 [file pharmaceutics-18-00506-s001.zip › pharmaceutics-4237718-supplementary.pdf]
